# Implementation of a new program of gestational diabetes screening and management in Morocco: a qualitative exploration of health workers’ perceptions

**DOI:** 10.1186/s12884-020-02979-9

**Published:** 2020-05-24

**Authors:** Bettina Utz, Bouchra Assarag, Touria Lekhal, Wim Van Damme, Vincent De Brouwere

**Affiliations:** 1 Department of Public Health, Institute of Tropical Medicine, Nationalestraat 155, 2000 Antwerp, Belgium; 2 National School of Public Health, Rabat, Morocco; 3 Service des Réseaux des Etablissements de Santé, Ministry of Health, Marrakech, Morocco

**Keywords:** Gestational diabetes, Screening, Primary health care, Motivation, North-Africa, Morocco

## Abstract

**Background:**

Gestational diabetes mellitus (GDM) is associated with an increased risk for a future type 2 diabetes mellitus in women and their children. As linkage between maternal health and non-communicable diseases, antenatal care plays a key role in the primary and secondary prevention of GDM associated adverse outcomes. While implementing a locally adapted GDM screening and management approach through antenatal care services at the primary level of care, we assessed its acceptability by the implementing health care providers.

**Methods:**

As part of a larger implementation effectiveness study assessing a decentralized gestational diabetes screening and management approach in the prefecture of Marrakech and the rural district of Al Haouz in Morocco, we conducted four focus group discussions with 29 primary health care providers and seven in-depth interviews with national and regional key informants. After transcription of data, we thematically analyzed the data using a combined deductive and inductive approach.

**Results:**

The intervention of screening and managing women with gestational diabetes added value to existing antenatal care services but presented an additional workload for first line health care providers. An existing lack of knowledge about gestational diabetes in the community and among private health care physicians required of public providers to spend more time on counselling women. Nurses had to adapt recommendations on diet to the socio-economic context of patients. Despite the additional task, especially nurses and midwives felt motivated by their gained capacity to detect and manage gestational diabetes, and to take decisions on treatment and follow-up.

**Conclusions:**

Detection and initial management of gestational diabetes is an acceptable strategy to extend the antenatal care service offer in Morocco and to facilitate service access for affected pregnant women. Despite its additional workload, gestational diabetes management can contribute to the professional motivation of primary level health care providers.

**Trial registration:**

clinicaltrials.gov; NCT02979756.

## Plain English summary

The rise of gestational diabetes prevalence worldwide presents an additional burden to health systems particularly in low resource settings. Gestational diabetes, a form of diabetes occurring in pregnancy, can already trigger the development of diabetes in the next generation and it is therefore key to test pregnant women in order to start treatment early. Antenatal care services provide a good opportunity not only to test for gestational diabetes but also to take care of women with this condition. A study conducted in Morocco revealed that health care providers face several challenges implementing integrated gestational diabetes testing and management into their services. Nevertheless, findings also showed that providers at the primary level of care perceived the activities being of added value to their antenatal care service provision. Despite the higher workload this additional task carried, nurses and midwives felt more motivated due to an increased recognition of their work by patients and a gain in autonomy to take decisions. The intervention showed that task-shifting for the detection and care of uncomplicated forms of gestational diabetes to primary level providers can be an important step in its management and in the prevention of future diabetes given that simple and clear algorithms exist and are combined with regular supervision.

## Background

Gestational diabetes mellitus (GDM), an elevated blood sugar for the first time detected in pregnancy, is gaining increasingly global health attention due to its rising prevalence worldwide with global estimates of 13.2% [1]. While highest overall rates of 23% are reported from South-East Asia [1], recent studies from Cameroon, Tanzania and Morocco already indicate prevalence rates of around 20% [2–4]. Given the impact of GDM on the health of future generations, the developmental origins of health and disease concept implies that early interventions already targeting the uterine environment of the unborn child can reduce the future risk for type 2 diabetes mellitus (T2DM) and other metabolic diseases [5, 6]. Early detection of GDM and its management can contribute to diabetes risk reduction in both the unborn child and the mother in the long term, and limit obstetric complications such as prolonged labor, shoulder dystocia and preeclampsia associated with GDM in the short term [7–10].

The role of primary health care (PHC) services is pivotal in controlling non-communicable diseases (NCDs) [11]. Antenatal care (ANC) can play an active part in primary and secondary prevention of GDM associated outcomes and thus act as a link between maternal health and NCDs. As such, it has the potential to assure a continuity of care for pregnant women with GDM and their children through the primary level.

In Morocco, pregnant women are requested to attend four ANC visits during the course of their pregnancy. A fasting blood sugar to screen for GDM is recommended in the first and second trimester by the nurses and midwives providing ANC at the health centers [12]. For these tests, women are usually referred to an external laboratory. Given the difficulty of access, severe delays in the timely diagnosis of GDM and its subsequent management prevail in Morocco [13].

To reduce such delays and to assess the feasibility and acceptability of GDM screening and management through ANC at the primary level of care, we conducted an effectiveness implementation trial [4]. The study included a qualitative component to explore the perceptions of health care providers and key informants towards GDM screening through ANC and the initiation of dietary management through primary care providers. Despite its potential to reduce delays, diagnosing and managing GDM at the primary level of care could have impacted on service provision. Therefore, our objective was to assess the experienced challenges of primary care providers and the views of key informants regarding GDM care provision through the primary level. In combination with the quantitative findings of this study [4], we hope to contribute to a comprehensive understanding regarding the potential benefits and challenges of adding GDM screening and management to the portfolio of current PHC activities.

## Methods

Between November 2016 and November 2017, we conducted a hybrid effectiveness-implementation trial to assess the clinical effectiveness of a GDM screening and management intervention and its implementation through first level health care providers [8]. Hybrid designs blend different research approaches and focus on the translation of research findings into clinical practice [14]. While GDM screening and management in the study intervention facilities followed clear algorithms based on the IADPSG/WHO criteria [15–17], national guidelines were applied in the control facilities. To explore opportunities and challenges of GDM testing and initial management through the primary level of care in Morocco and thus assess the feasibility of such an intervention, we conducted interviews and discussions with providers of the study facilities and with key informants**.**

### Study setting

The study was carried out in 20 randomly selected PHC facilities from a list of health facilities in the districts providing at least 30 ANC consultations each month. Ten health centers were located in the district of Marrakech, a predominantly urban area and ten facilities were located in the neighboring district Al Haouz, which is mainly rural and mountainous. Both districts are part of the wider region of Marrakech-Safi and comprise of a total population of 1.9 million with 92 health centers and 53 dispensaries providing PHC services to the population [18]. The study facilities were equally distributed into urban/peri-urban and rural health centers. Although the service package offered in the public health centers did not differ between urban and rural locations, access to some of the rural sites was geographically limited.

### Study participants and sampling

Between January and March 2017, we conducted four focus group discussions (FGD) with the health care providers in charge of ANC and GDM screening in all selected study health centers. Both ANC providers trained in GDM screening and management from the intervention sites were invited, while two providers in charge of the ANC services from each control health center attended. Overall, 15 providers from the ten intervention health centers and 14 providers from the nine control facilities took part in the FGDs. Each FGD was conducted with between five to nine participants and separate focus groups were led with providers from control and intervention sites in both study districts. In addition, we did seven interviews with key informants including clinicians involved in diabetes care (2) as well as program managers (public health/ maternal health/ NCDs) on national (2) and regional/district level (3).

### Data collection

FGDs took place in a quiet room at the district/provincial directorates and were led by an experienced female moderator (BU, BA) and a research assistant taking notes. Interviews with key informants were conducted at the offices of the interviewees by BU and BA. Pre-tested topic guides were used to explore opportunities and challenges for GDM screening and management, and to assess perceptions about the feasibility of such an intervention as part of the existing service package at the primary level of care. Topics selected for discussion included the role of antenatal care to screen for GDM, providers views and their experience with women’s attitudes towards detection and management of GDM, the influence of such a GDM activity on their work and the role of communication with other actors. The topics for the questions were derived from the challenges identified by providers during a situational analysis conducted prior to this trial [13]. Mean interview duration was 39 min (min 17; max 80) and FGDs lasted on average 95 min (min 80; max 100). Before each interview and FGD, participants were requested to read the provided information letter and give their written informed consent. All interviews and discussions were conducted in French, digitally recorded and later transcribed ad verbatim. If some respondents switched during the interviews into Arabic, the bilingual transcriber, a qualified translator, translated these passages into French. The correctness of the transcription was cross-checked by BA on a sample of transcripts.

### Data analysis

All French transcripts were transferred into NVIVO software version 10. The authors who conducted the interviews also analyzed the data. After repeatedly reading all interview transcripts and to familiarize with the data, information was subsequently arranged into codes. Coding was done in a combined inductive and deductive approach, with the deductive coding oriented at the interview topic guide, while open coding was used for new emerging codes in an inductive way. Codes were then grouped into categories and overarching themes. Coding of all scripts was done by one researcher (BU), with intermittent cross-checking of scripts by BA. Categories and emerging themes were discussed by the two researchers involved in data analysis (BU and BA). Translation of the quotes, codes and categories from French into English took place after all data was analyzed (BU). Without summarizing or removing content, data was translated word by word, while assuring its original meaning. If translation revealed several options, the most suitable meaning was validated by the second researcher who was not directly involved in data collection nor analysis (VDB). Both researchers involved in translation are bilingual and have extensive contextual experience in Morocco.

## Results

The mean age of FGD participants was 41 years and their average professional experience 13 years. Most participants were female (93.1%), nurses and midwives represented the majority of participants (Table 1). Key informants were senior program leaders at national and regional/district level working in the field of diabetes and/or maternal health (*n* = 5) and clinicians involved in diabetes care at referral level (*n* = 2). Our results revealed the challenges providers face when screening and managing GDM but also the motivational leverage such an additional task can provide. The following themes emerged from the data: acceptability and accessibility of screening, importance of communication and collaboration, challenges with the management of GDM, need for service re-organization and gain in motivation (Table 2).

**Table 1:** Socio-demographic characteristics of FGD participants

Facility type	Intervention	Control	Total
No. of FGD participants, *n*	15 (51.7)	14 (48.3)	29
Mean age, years [min-max]	42 [28,56]	39 [25,55]	41 [25,56]
Average professional experience, years [min-max]	14.9 [4,36]	11.3 [0.25–25]	13.1 [0,25–36]
Nurses, *n* (%)	7 (46.7)	7 (50)	14 (48.3)
Midwives, *n* (%)	4 (26.7)	3 (21.4)	7 (24.1)
Doctors, *n* (%)	4 (26.7)	4 (28.6)	8 (27.6)

**Table 2:** Themes, categories and codes

Themes	Categories	Codes
Acceptability of screening at health centre	Disease perception	Fear of diabetes complications
		Fear of insulin
		GDM not considered diabetes
		Stigma
	Provider choice	Preference of the private sector
	Communication barrier	Use of diabetes terminology
	Service barriers	Extra workload
		Waiting times
	Provider attitude	Welcoming
		Familiarity with provider
	Added value to existing service	Increasing value of ANC
		Availability of tests in facility
	Demand induced	Increasing demand for testing
	Time gain	Organised referrals
		Reducing delays of external testing
	Reducing expenses	Reduced/no costs for tests
Accessibility of testing	Service availability	Testing material in place
		Providers trained
		IEC at health centre
	Geographical accessibility	Short distance to the health facility
		Transport available
	Financial accessibility	No expenses for testing
		Transport affordable
	Cultural accessibility	Pre-defined role of women
		Lack of decision making power
		Household responsibilities
	Testing preconditions limiting	Consent
		Fasting
		ANC timing
Management challenges	Diet	Extra expenses for food
		Adaptation to local food
		Isolation through diet
		Time required for counselling
	Medication (Insulin)	Fear of insulin
		Under-prescription
		Unavailability
	Material	Test-strips for self-testing expensive
		Diet brochures not adapted
	Referral	Delay in getting appointments
Communication/ Collaboration needs	Transparency of providers	Provision of information & education
		Counselling of family members
	Use of mobile phone	Linkage to specialist
		Used for follow-up
	Husband and family support	Acceptance of diagnosis
		Adherence to follow-up
	Meeting peers	Exchange of experience
		Anxiety reduction
		Information on diet
		Feeling of belonging
		Provision of support
		Exposure to positive examples
	Private sector involvement	Different diagnostic thresholds used
		Conflicting information provided
	Sensitization	Need for more training
		Importance of the role of media
		Information on prevalence
		Raising awareness of treatment
		Including postpartum testing
Gain in motivation	Professional gains	Knowledge
		Autonomy
		Decision making
		Performance
		Empowerment
		Teamwork
	Patient acknowledgement	Trust
		Patient-provider relationship
		Recognition
	Personal gains	Self-esteem
		Responsibility
Service re-organization	Re-organisation	Limiting number of tests
		Organising additional sessions
		Task-shifting
		Integration into existing service
	Constraints for screening integration	Documentation need
		Service interruption
		Extra workload
		Lack of clarity about continuation after study

### Acceptability of screening

We identified three issues in relation to the acceptance of screening: the perception of the disease by the patients, as well as enabling factors and barriers to screening at provider level.

Having a ‘disease’ in pregnancy is difficult to accept and might be a reason not to return for further ANC visits because of the anxiety associated with not being healthy. For some women the diagnosis was shocking. Stigma associated with the term diabetes relates to the association of ‘diabetes’ with a life-long handicap, the need for insulin or fear of complications in relation to pregnancy.

*"She cried- a woman with a history of a child who died and another child who is sick. When I told her she has gestational diabetes, she cried.” (FGD participant, control site)*

Therefore, the use of terminology is very important and can reduce not only fear and stigma associated with the term ‘diabetes’ but also places gestational diabetes in the right context. Some of the nurses told us that they avoid using ‘diabetes’ in their explanations.

*"Most of the time I do not pronounce the word diabetes. I tell her 'you have a sugar level that is a bit high. A pregnant woman should not have such a level [ … ].” (FGD participant, control site)*

Given the high prevalence of diabetes in Morocco, some women already have diabetic relatives. Being familiar with high blood sugar levels of diabetic family members, the lower values for the diagnosis of gestational diabetes (0.92 g/l), gave them a false sense of security.

*“For them 1g is nothing, because they have diabetics in their family. When one has 1g, 1.5g that’s nothing at all [ … ]. So, they left and consulted others, specialists.” (FGD participant, control site)*

Perceived barriers to screening included waiting times for testing and the perceived long procedure of the oral glucose tolerance test (OGTT). Providers mentioned however that raising awareness about GDM increases demand for and acceptance of screening. Nurses of the intervention sites stated that offering extended services in the health centers, including free tests for GDM screening, not only reduce delays, but also attract more women to ANC and increase client satisfaction.

*"Before: the woman came: just weight, height. Now, she is aware that something is being done for her and she is satisfied with the service she is given.” (FGD participant, intervention site)*

Respondents also mentioned that screening at the health center is more acceptable to women, as ANC providers are often already familiar with the women through current or previous pregnancies.

### Accessibility of testing

Availability of screening services, but also geographical, financial and cultural factors determine access to testing. Particularly at the rural sites, costs for transport to the health facility impaired accessibility. Lack of decision-making power implied that some women first had to get the permission of their husbands before being tested.

*“Especially in our population it is the husband who decides for the woman, and the mother in law who decides for her, if she can go and do her blood tests or not.” (FGD participant, intervention site)”*

Not all women were fasting when attending ANC and had to return for another screening appointment. Some women visited the health center only very late in pregnancy thus limiting the possibility to be screened for GDM.

### Communication and collaboration needs

Providers, but also family and peers play an important role in the support of women diagnosed with GDM. Communication and collaboration within and between these groups are essential for a positive GDM response in addition to the sensitization of the population. Husbands and family in particular play a crucial role for the acceptance of the condition and for assuring follow-up. Several respondents mentioned the importance to involve family members in counselling.

*“The woman tells me ‘you have to explain it to my husband’ because the husband told her ‘you are not diabetic; these values are not high’ [ … ]. Therefore, I have to explain her husband why he needs to bring her to attend the follow-up.” (Key informant)*

In addition to family support, providers highlighted that women benefit from peer support by other women with GDM. Therefore, some of the providers organized follow-up in their services in such a way that affected women, including newly diagnosed, could meet and exchange experiences.

*“[ … ] She will speak about her experience, she will speak about her failures and successes [ … ] It is extremely motivating for other people to listen to this patient and ask practical questions. [ … ] When a doctor or a nurse speaks, they still remain educators, he or she did not experience gestational diabetes [ … ].” (Key informant)*

Respondents highlighted the need for transparency in communication with the women, to provide education and inform them about the meaning of their glucose values. Nurses and midwives often used their mobile phones to communicate with the women if further questions arose but also to make appointments with specialists if needed.

The perceived lack of knowledge about gestational diabetes in the general population would require sensitization about GDM in general and the possibility to be tested. GDM is not covered by the media which was considered by one health care provider a missed opportunity in support of their educational tasks.

*"If the media plays its part, it is much easier. If people knew what gestational diabetes is, the risks and everything, we could have services such as those we have recently for breast cancer. Women come alone to ask for screening, because they heard that on TV.” (FGD participant, control site)*

The lack of knowledge is not only confined to the population. Even health care providers have limited information about gestational diabetes and latest diagnostic criteria. Often, they use the same diagnostic thresholds for gestational diabetes as for diabetes. This was reported for women who attended the private for-profit sector or other health facilities not included in the study. The advice given there stood often in contrast to the information women received in the study health facilities.

*“It's a problem with the private [sector]. [ … ] When she consulted the private [provider], she had a blood glucose of 1,1 [g/l] and they tell her ‘You have nothing. They are doing a study, they lie to you to earn money [ … ]’. They disgrace us and we are in conflict with each other. And this is not good, because public health cannot do everything and idem for the private sector. We have to be complementary.” (FGD participant, control site)*

However, a disagreement can also be an opportunity for behavioral change. Informal conversations with nurses of some of the health centers during our supervision revealed that several private providers around their centers started diagnosing women with GDM based on a fasting glycemia level of 0.92 g/l which was not the case before. This change in practice originating in the public sector and being taken up by private providers is a positive development that reaffirms nurses working in the public centers.

*"By creating conflict, one creates the truth. [ … ] It will simply result in the change of attitude of this [private] physician towards reality [ … ]. He will go and look at the topic of gestational diabetes. [ … ] Maybe the doctor will oppose once or twice. But considering the frequency of gestational diabetes he will realize that the nurse or the doctor or the midwife at the health center is right. So, it's maybe positive that this way of managing [GDM] will exceed the public sector and reaches even the private [sector]."(Key informant)*

### Management challenges

Providers mentioned the adherence to nutritional advice as one of the management challenges. After a woman is diagnosed with GDM, the first treatment usually consists of nutritional therapy coupled with physical exercise. Nutritional counselling including information on food preparation and how to integrate the diet into the family meals needed particular consideration.

*“To ask her to eat alone in front of the family, of her husband, depresses her. For example, when you ask her to eat alone, her husband loses his respect for her, even the mother-in-law. [ … ] It is better to ask her what she eats and adapt her diet to that and let her eat with her husband and children.” (FGD participant, intervention site)*

For the purpose of this study, the Ministry of Health developed for women with GDM a brochure on nutrition. However, nurses in rural areas found its recommendations particularly difficult to follow, because these did not consider financial constraints to purchase certain food items. Therefore, health care providers had to adapt recommendations to the socio-economic and local context of patients.

*“When I explained to her what she was going to eat she was surprised. She told me she will be hungry. This was a poor woman, she had conflicts with her husband, conflicts with her mother-in-law and no means [ … ]. We asked her what she normally eats to try and advise her a diet that was adapted to what was available for her and according to her means without the need to buy more items; step by step with the food she uses at home.” (FGD participant, intervention site)*

Despite the difficulties for some women to follow a diet, the majority tried to adhere because they fear insulin. This was the card played out by some nurses to make women adhere to the diet.

*“We insisted that they do physical exercise and follow a diet to avoid proceeding to a treatment with insulin. We explained to them that ‘otherwise you are obliged to be treated with insulin’. This has pushed patients to adhere.” (FGD participant, control site)*

In Morocco, only insulin is currently approved as treatment for women whose gestational diabetes is not sufficiently controlled by diet alone. However, not all doctors feel comfortable with insulin prescriptions.

*"I think what is worrying and what scares the caregiver is to manage also insulin. Is it the patient who is afraid of insulin or is it the doctor who is afraid of insulin? So, it leads to this therapeutic inertia. [ … ] So, if metformin is available, the management is more flexible, it will make things easy." (Key informant)*

### Service re-organization

Adding GDM screening and follow up to the already full work schedule of ANC providers often needed some re-organization of services in the intervention facilities. To accommodate GDM as an additional activity in their ANC schedule, some providers limited the number of women for GDM screening and follow-up and organized additional sessions for testing.

*“I tried to make a planning. For example, I do one or two sessions a week. I note the names of the women I have summoned, for example 5 to 6 women per session, just to limit this problem. Because really, it coincides with my other daily activities [ … ].” (FGD participant, intervention site)*

In the discussions, the issue of workload and task shifting came up several times. Some providers reported problems to delegate their task of GDM screening and follow-up when on leave, as only two providers per center (nurse/midwife and doctor) were trained in the intervention.

*“Someone must be able to work in my place. If I get sick or go on leave, the program stops. [ … ]. It is necessary that everyone knows how to conduct this activity, not a single person in the center. If she is not there, everything stops.” (FGD participant, intervention site)*

Given the additional tasks, including increased documentation requirements, most respondents underlined the importance of GDM screening and management training of all providers involved in providing care to pregnant women.

### Gain in motivation

All the above-mentioned aspects reflect the extra burden of this activity for ANC providers in terms of time management and service organization. Nevertheless, the motivational effect of incorporating GDM screening and management came up as an important facet of this intervention.

Providers highlighted that screening and regular follow-up improved trust and their relationship with the patients.

*“This study gave great importance and value to our ANC, because the women do not only come to measure uterine height, weight and do ultrasound. There is an additional activity that strengthens the relation with the woman, because she spends the entire morning at our center with the midwife.” (FGD participant, intervention site)*

ANC providers felt motivated and valued by their patients and gained in esteem and recognition for their provided services.

*“Really, I feel useful doing something with these women … [ … ]. I feel that on a personal level I learnt new things I did not know before. I acquired additional information. Really this affects even your relationship with the woman. You have more confidence. You offer her something you did not know about before. [ … ] When you speak with the woman at ease and you provide her with useful information, she trusts you, she asks you questions. At this moment, in this situation you feel that you are useful and motivated to do more things despite the workload and all that.” (FGD participant, intervention site)*

Another important aspect underlined by several nurse-midwives in the intervention facilities was a gain in autonomy and the strengthening of their role in the center.

*“I felt that the role of a midwife is no longer limited to making the detection of a pregnancy at risk, to leave the decision making to the doctor, [that] I work and do the paperwork and then the doctor takes the decision. I liked it that it is me now to take the decision.” (FGD participant, intervention site)*

This autonomy affected also the doctors of the health center as they felt more involved in decision making.

*“It is not only the midwife who thinks that she became important, even the general practitioners realized this. When they found a pregnant woman with a GDM they referred her. Now we take a decision [ … ]. We begin to conduct searches, we make contact with the endocrinologists, they tell us what to do. We have the impression that we became half-endocrinologists. It is motivating. [ … ] And the women are satisfied [ … ]” (FGD participant, intervention site)*

Finally, as one respondent puts it, all is about working as a team in the health facility to provide the best services to the patients.

*“It is important [ … ] that everybody finds one’s place and that the medical doctor doesn’t think that the nurse or midwife takes his place. She completes his actions and they must work with a team spirit.” (Key informant).*

## Discussion

Discussions with health care providers and key informants revealed that GDM screening and follow-up through the primary level of care in Morocco is an acceptable strategy, although it is facing several challenges. These include not only access to and acceptability of services, but extend to communication and collaboration issues as well as challenges with management and service organization. Nevertheless, our findings also indicate the potential of such a strategy in increasing the motivation of providers, which may counteract the perceived barriers that will be discussed in the following sections.

Accessibility and acceptability besides the availability of services are known bottlenecks to the utilization and coverage of services and have already been described by Tanahashi in his framework on the capacity of health systems to provide care [19]. Nevertheless, disease perception and a negative connotation related to the term “diabetes” requires particular attention of health care providers to a sensitive communication with patients and family members.

Lack of public sensitization of pregnant women about GDM increases the responsibilities for ANC providers to inform and educate women. Health professionals are often not familiar with GDM and have no notion of the difference between diabetes and GDM. This lack of knowledge particularly affected the collaboration of trained providers with the private sector and led to controversies in recommendations. The dilemma between the often unregulated private and the public sector and how to overcome this gap has already been described elsewhere [20]. However, our results pointed out that private sector providers adopted some of the public sector practices after initial resistance. This indicates that both do not work in isolation and that changes in the public sector can influence private sector practice. However, more public sensitization on GDM as well as training of providers at all levels need to be applied across the public-private sector lines in order to generate an aligned approach to GDM screening and management in Morocco.

Initial management of GDM consists of dietary counselling, and providers had to adapt their nutritional advice to the socio-economic background of women. Especially, counselling women from poor households requires a certain amount of flexibility in recommendations, as some women cannot afford specific food items listed in the nutritional guidelines. Respondents in our study underlined the importance of incorporating the nutritional changes into family meals instead of adhering to a diet in isolation. Involvement of the family in lifestyle changes may not only improve better adherence and diabetes control of patients, but also result in better health of family members as studies related to diabetes type 2 have shown [21]. Particularly, nurses and midwives have a core function in health education [22]. Thus, their access to women who are in charge of their family’s nutrition opens a unique opportunity to counsel about a healthy lifestyle that is not only focused on the individual. Therefore ANC, the entry point of many women to health care, could play an increasing role in general health education and in the prevention of diabetes and other NCDs.

Nevertheless, adding more activities to the already high workload of primary care providers may be counterproductive and result in a reduction of the quality of services [23]. Our findings revealed that the additional workload at the intervention sites often resulted in a provider-led re-organization of ANC services to account for the additional work charge of testing patients. However, service re-organization needs to be coupled with training of all members of ANC staff to enable a continuity in service provision if GDM screening and management would become integral part of ANC. The combination of practice re-organization and workforce development can furthermore have a positive impact on access to these services [24].

Delegation of tasks, an important aspect in the theme service organization, is possible in chronic disease management in low-resource countries, given that nurses are trained, have clear instructions to perform repetitive tasks and are supported by regular supervision [25]. Nurse-led diabetes management following protocols showed similar results when compared to care provided by doctors and was accompanied by a higher patient satisfaction [26, 27]. The latter is an important aspect, as task shifting is often narrowed down as the only solution to workforce shortages. Client satisfaction is relevant for adherence to treatment and follow-up [28]. Empathy of providers has shown to increase satisfaction levels and compliance [29]. GDM diagnosis is often accompanied by patients’ fears [30], and therefore requires a high degree of empathy to reduce anxiety. Studies have shown that nurses have a high capacity to provide empathic care [31, 32]. Given their central role in the follow-up of uncomplicated pregnancies, nurses and midwives are therefore ideally positioned to comprehensively care for GDM affected women [33].

Our findings revealed that a gain in motivation played an important role for the acceptability of the intervention. This has been reflected in a reported increase in self-esteem and recognition through peers and patients. According to Maslow’s hierarchy of needs describing the causes for motivation in an ascending order [34, 35], the intervention affected the three upper levels of a five-tier pyramid; social belonging, self-esteem and self-actualization. The gain in autonomy in performing the activities and taking decisions increased provider’s intrinsic motivation, a phenomenon which is integral part of the self-determination theory [36]. Furthermore, according to the diffusion of innovation model, an intervention is likely to be adopted, if it responds to a perceived need, has observable benefits, is simple to use, can be tried out (trialability) and adapted to local requirements [37]. This intervention appeared to meet these requirements, as testing at the primary level of care was perceived as an advantage by enlarging ANC service offer, was simple to use, had benefits in terms of a reduction in delays in testing and management and gave providers the liberty to adapt the activity to their schedule and to patients’ availability.

Nevertheless, our findings have to be interpreted with caution as they were introduced as a short term, study related intervention. However, the results indicate that involving ANC providers in a new intervention broadening their offer of care and extending their decision-making autonomy within clear limits may not only increase the pride associated with their role, but also positively affect the trust between providers and patients. Although the additional task adds to the already high workload of health professionals, this seems to be counterbalanced by the gain in motivation. To sustain such motivation, official recognition to reward employees’ efforts is a further step [38]. This has also been shown in a study from Tanzania indicating that recognition and appreciation of PHC providers are important to sustain motivation [39].

### Limitations

Health care providers at both intervention and control sites were involved in data collection for the study and received a small remuneration (equivalent of 20€/month) for the additional paperwork. Critics may consider this as incentive that may have influenced motivation of providers. However, the amount is relatively low in the Moroccan context and it can be doubted that such a small sum alone would have increased motivation. A further limitation to the findings is the possibility of social desirability bias during the interviews but our experience during regular supervision visits and information received from patients support the reported findings. Our results reflect only the views of a limited number of health care providers involved in this implementation trial. Therefore, they may not be generalizable to other settings. Besides, there may be translation related limitations, as concepts in French may have another meaning in English (conceptual equivalence). Some meanings may have been lost in translation, although ambiguous words were cross-checked and validated by the bilingual researchers involved in translation. While the authors own identity may have affected the analysis, researchers addressed this potential source of bias by being attentive to their own assumptions (reflexivity).

## Conclusions

In the Moroccan context studied, introduction of GDM screening and initial management may be well accepted by affected women and staff alike, given that the challenges regarding its acceptability, communication and collaboration requirements and the implications for service re-organization are adequately addressed. Despite the additional workload such activity can act as motivational leverage for PHC providers and might serve as example for a feasible new strategy to extend the service offer in ANC. Further research is required to explore if and how such interventions can sustain motivation in an environment that is continuously facing staff shortages and high individual workload.

## Data Availability

All data generated or analysed during this study are included in this published article. Datasets used are available from the corresponding author on reasonable request.

## Abbreviations

ANC: Antenatal Care
FGD: Focus Group Discussion
GDM: Gestational Diabetes Mellitus
NCD: Non-Communicable Disease
OGTT: Oral Glucose Tolerance Test
PHC: Primary Health Care
